# Closing in on Chemical Bonds by Opening up Relativity Theory

**DOI:** 10.3390/ijms9030272

**Published:** 2008-03-12

**Authors:** Cynthia Kolb Whitney

**Affiliations:** 141 Rhinecliff Street, Arlington, MA 02476-7331, USA, E-mail: Galilean_Electrodynamics@comcast.net

**Keywords:** Chemical Bonds, Ionization Potentials, Quantum Mechanics, Special Relativity Theory, Two Step Light Theory

## Abstract

This paper develops a connection between the phenomenology of chemical bonding and the theory of relativity. Empirical correlations between electron numbers in atoms and chemical bond stabilities in molecules are first reviewed and extended. Quantitative chemical bond strengths are then related to ionization potentials in elements. Striking patterns in ionization potentials are revealed when the data are viewed in an element-independent way, where element-specific details are removed via an appropriate scaling law. The scale factor involved is not explained by quantum mechanics; it is revealed only when one goes back further, to the development of Einstein’s special relativity theory.

## 1. Introduction

Can there exist any plausible connection between the phenomenology of chemical bonding and the theory of relativity? This paper argues in the affirmative. It stands as testament to the idea that the disciplines of Chemistry and Physics need to exist in intimate symbiosis with each other. Chemistry produces reams and reams of interesting data. Physicists should revel in that data: chemical experiments are far more feasible to conduct than cosmological ‘experiments’, and chemical data is far less costly to acquire than elementary-particle data. Chemistry needs Physics too, though perhaps not as much, to help infer simple laws from complex data.

The argument here begins with some comments on the Periodic Table. In Sect. 2, known empirical correlations between electron counts in elements and chemical bond stabilities in molecules are reviewed, and then extended beyond the familiar situations.

The argument then moves to a more quantitative domain. Clearly, the quantitative strengths of chemical bonds in molecules must reflect the same physics as do the quantitative magnitudes of ionization potentials of elements. The understanding of that physics is presently based on quantum mechanics (QM), and that understanding is very incomplete. Sect. 3 shows striking patterns in ionization potentials that are revealed only when the data are viewed in an element-independent way, where element-specific details are removed via an appropriate scaling law. The required scale factor is *M* / *Z*, where *M* is nuclear mass and *Z* is nuclear charge.

The need for the *M* / *Z* factor is not explained within QM. It is revealed only when one goes back further, to the development of Einstein’s special relativity theory (SRT). It is an unfortunate accident of history that the development of SRT preceded the discovery of much of the phenomenology that drove the development of QM. Had the historical sequence been reversed, both theories might have developed differently. Sects. 4 and 5 show how.

Sect. 4 introduces a variant version of QM, which includes known results about atoms, but also offers alternative interpretations and simpler calculation approaches, one of which accounts for the *M* / *Z* scaling. The source for this theory is a covering theory for SRT, which includes Einstein’s SRT, along with some additional information, some of which leads to the different point of departure for QM used in Sect. 4. Sect. 5 summarizes this covering theory, known as ‘Two-Step Light’.

## 2. On the Periodic Table and the Chemical Bonds

Since the time of Mendeleev, the Periodic Table (PT) has been the fundamental organizing tool of Chemistry. Its history of development, and our present understanding of its significance, are documented Eric Scerri in [1]. The PT has been partially rationalized in modern times, with the development of QM. As one progresses through the elements, more and more electrons are involved in a multi-electron state that is envisioned as a product of single-electron states like the states that QM attributes to the prototypical Hydrogen atom. These single-electron states are characterized by quantum numbers, and there is some order to the way in which available quantum numbers enter into the mix. But the nominal order is not really understood, and anyway there are violations to it: about 20% of elements depart from the prevailing pattern for filling available quantum states.

Not being fully satisfied with understanding of the PT has driven many individuals to develop different display formats for the PT. The goal of organizing the elements according to chemical properties, like valence, has produced the wheels, spirals, helixes, three-dimensional pretzels, conics, trees, and more, collected and richly displayed by Spronsen [2]. The goal of organizing the elements according to shell structure or electron configuration has produced some more linear types of displays, which are generally favored by Mazurs [3].

The historically numerous revisits to the PT do express the strong tradition in science for looking at the same, known, information again and again, but arranged in a variety of different ways. This sort of exercise is important for scientists to do, and *keep* doing, regularly. Experience has shown over and over that looking at any information from a different angle can reveal aspects of that information not consciously noted before, and so can trigger new and interesting insights and new questions to ask.

**Figure 1.**
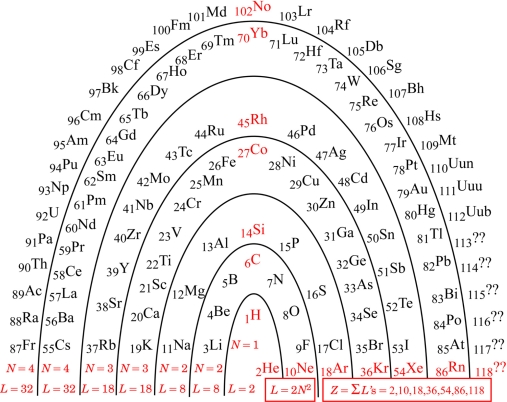
The Periodic Arch (PA).

I am no different from other authors: I too have been driven to understand better, and have developed a favored way of picturing the PT. The original conception had elements arranged in columns for chemical similarity. I like the idea of an architectural metaphor, but not that particular metaphor. As it has accommodated more and more newly discovered elements, the columns have come to include some very short ones to the center left, where the Lanthanide and Actinide series must go. Being unable to support any metaphorical ‘roof’ of all-encompassing understanding, those columns are generally stored away in the ‘basement’ (footnotes), to be remembered and retrieved as needed by the user. My preferred remedy for the situation is to change the architectural metaphor: instead of thinking ‘columns’, think ‘arch’. Figure 1 shows the ‘Periodic Arch’.

Any architectural metaphor naturally attracts one’s attention to the idea of a ‘foundation’. The foundation of the PA is the red information along the bottom of the arch: the *N* parameter, the arch layer lengths *L*, and the noble gasses and their atomic numbers *Z*_noble_ :

(1) $$L=2N^{2}\text{ for }N=1,2,2,3,3,4,4,...\;,\;Z_{\text{noble}}=\sum_{N}L(N)=2,10,18,36,54,86,118,...$$

Although actual element discovery is presently only up to *Z* = 112, one can anticipate that the pattern identified will be followed by any heavier elements that may be found in the future. Should we ever reach and progress beyond *Z* = 118, we will be into the regime of *N* = 5 and 2*N*^2^ = 2(5)^2^ = 50, and so on, according to the pattern.

While the pattern, 2*N*^2^ for *N* = 1,2,2,3,3,4,4,..., was also detectable in the rows of the traditional PT, it was rather hidden there because of the confusing footnotes for insertions. Mingos [4] noted the numbers, and described them by an algebraic formula (numerically equivalent though formally different), but did not pursue a deeper meaning. Siekierski & Burgess [5] discussed in some detail the believed reasons why each atom turns out the way it does, but not why the overall pattern is what it is.

The arch metaphor further draws one’s attention to the idea of ‘keystone’. The keystones in the PA are the red elements up the middle of Fig. 1: starting with Hydrogen, and above it Carbon, Silicon, Cobalt, Rhodium, Ytterbium, and Nobelium.

The foundation algebra and the keystone elements of the PA turn out to be truly useful for making an initial qualitative comment on chemical bonds in this Section. Hydrogen turns out to be further useful for the subsequent quantitative analyses of ionization potentials in the next Section.

We know that some molecules tend to be stable, whereas some tend to be highly reactive. For or example, some simple dimers, like H_2_ NaCl, *etc.*, may escape into air, or dissolve and dissociate in water, but they will not react explosively without a spark or catalyst of some kind. But some monomers, like atomic Hydrogen and metallic sodium, are more risky.

The present understanding for these simple cases is often phrased in terms of ‘complete’ and ‘incomplete’ electron ‘shells’, which are said to surround atoms. But what should be said about larger molecules, with many atoms in them? Evidently, molecules that are relatively stable must have strong chemical bonds throughout, and molecules that are strongly reactive must have some weak chemical bonds somewhere. In order to extend the somewhat limited ‘shell’ understanding to larger molecules, consider the following more general candidate statement:

**Proposition 1:** Molecules that are relatively stable have total electron counts such that every atom present can be assigned an electron count equal to that of a noble gas, or else zero.

The proposition implies redistribution of electron resources. The idea is that electrons are like cash money: totally fungible – even more so than cash money, since they have no serial numbers or other individually distinguishing characteristics.

The reader can readily verify that Proposition 1 is satisfied by the simple dimers like H_2_, NaCl, *etc.*, as well as water H_2_O, CO_2_, and many other simple trimers. Table 1 gives a half dozen of the increasingly complex other examples that prompted the articulation of Proposition 1. There exist many more examples; there are, for example, many hydrocarbon fuels, and so far all of them examined do have electron counts that can be redistributed in the way that Proposition 1 describes. Maybe the stability thus implied is why the hydrocarbon fuels are commercially viable.

Observe in Table 1 that the noble-gas number (or zero) assigned to each atom is generally for the noble gas (or zero) as close as possible to that element. Observe that the ‘keystone’ elements are all special, being equidistant from *two* noble gasses, or in the case of Hydrogen, equidistant from a noble gas and a priestly-class 0. An equidistant condition allows two choices of electron reassignment.

The equidistant condition of Hydrogen perhaps explains something mysterious observed in deep space. Evidently, any Hydrogen atom would want either to form a Hydrogen molecule (2 electrons total), or if that were not possible, then to dissociate and form plasma (proton with no electron). Plasma is indeed frequently observed in deep space. The amazing possibilities for Carbon-Carbon bonds are well known (chains, rings, sheets, tubes, balls…life…), and seem likely to trace to its special equidistant condition. Silicon is known to be very similar to Carbon, and the other keystone elements may also turn out to be more similar than is presently recognized.

Sometimes, the condition specified in Proposition 1 cannot be achieved. For example, it cannot be achieved for any molecule that has an electron count that is an odd number. Also, it cannot be achieved for some atmospheric gasses, such as Oxygen O_2_, Ozone O_3_, or Nitrous Oxide NO. These molecules are often rather highly reactive. These situations prompt one to consider a second candidate statement that is the converse to Proposition 1:

**Proposition 2:** Molecules that are highly reactive have total electron counts such that *not* every atom present can be assigned an electron count equal to that of a noble gas, or else zero.

The conclusion to be drawn from this preliminary qualitative analysis is that chemical bonding has less to do with pair-wise connection between atoms, and more to do with molecule-wide collective status of all atoms. Strong bonding is about assigning electrons in such a way as to promote every atom present, either to the status of ‘noble gas’ (possessing a comfortable number of electrons), or else to the status of ‘priestly class’ (needing no worldly electrons at all). If that goal is achieved, then the molecule has a population of well-satisfied atoms, constituting a relatively stable society.

## 3. Patterns in Ionization Potentials

It is desirable now to strive for more quantitative assessment of chemical bonds. But molecules are very complex, and it is rational to start with the constituent atoms. Atoms have ionization potentials (as scalar variables, *IP*’s), which are analogous to energies of chemical bonds: *IP*’s represent the strength with which electrons are bound to atoms. Data are generally available for several ionization orders (as integer variables, *IO*’s) of most elements. It is a rich database to explore.

**Figure 2.**
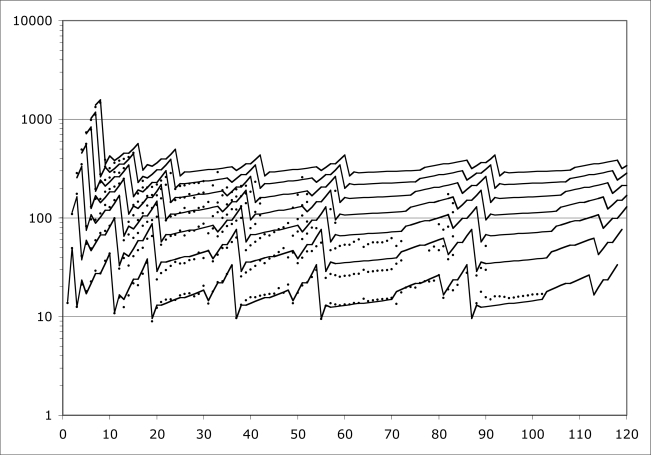
Ionization potentials, scaled appropriately and modeled algebraically.

Even the *IP*’s of atoms are not yet generally understood as well as they need to be. I have been studying the problem for some years, and Fig. 2 expresses my current best understanding of it. The Figure depicts the behavior of *IP*’s for all elements (nuclear charge *Z* = 1 to *Z* = 120 shown). Element *Z* actually allows *Z* ionization potentials, but for larger *Z*, many *IP*’s are not so easy to measure. Readily available data go only to seventh order, so that is how many orders are shown here.

The points on Fig. 2 are measured *IP* electron volts, scaled for comparison with each other as indicated by a new theory summarized in subsequent Sections of this paper. The scale factor is *M* / *Z* where *M* is nuclear mass number. This scale factor is in no way indicated by traditional QM.

The lines on Fig. 2 represent my algebraic model for *IP*’s, rendered in its current best state of development. The model is capable of producing plausible estimates for all *M* / *Z* -scaled *IP*’s for all *IO*’s, even beyond those measured, and all *Z*’s, even beyond those known to exist.

The model-development approach is called ‘data mining’. Figure 2 has less than 400 out of approximately 5000 desired data points. But that is enough data points to support the development of the algebraic model.

The work involved is a good example of continuing positive feedback between theory and experiment. Theory shows what to look for; experiment shows what to try to understand.

The first development step was fundamentally observational: for *IO* = 1, with *M* / *Z* scaling, there are consistent rises on periods, and consistent mid-period similarity to Hydrogen (*Z* = 1). For *IO* > 1, there is consistent scaling with *IO*. There are several ways that the scaling can be described, and the simplest way found so far is previewed as follows:

1. First-order *IP*’s contain ALL the information necessary to predict ALL higher-order *IP*’s via scaling.
2. Every ionization potential *IP* of any order *IO* can be expressed as a function of at most two first-order *IP*’s.
3. For a given ionization order *IO* > 1, the ionization potentials for all elements start at element *Z* = *IO*, and follow a pattern similar to the *IP*’s for *IO* = 1, except for a shift to the right and a moderation of excursions.

Details follow.

For a given value of *IO*, the first element that has an *IP* value for that *IO* is the element with nuclear charge *Z* = *IO*. This *IP* represents completely stripping all the electrons from the atom, thus leaving an ion fully charged to +*IO*. The *IP* is given by

(2) $$IP_{IO,IO}=C\times IP_{1,1}\times IO^{2},$$

where *IP*_1,1_ is the one and only ionization potential for atomic Hydrogen, and *C* is a constant factor. In previous incarnations of this work [6,7], I expressed the opinion that this factor, like so many others revealed shortly in the model, involved a 7, and some 2’s, and therefore had to be 7 / 4. I saw that the data were slightly off from 7 / 4, and attributed that discrepancy to experimental error. I was wrong. I am now sure that the factor is not 7 / 4; it is 2, exactly, without any discernable experimental error in the data. *Mea culpa*. The ‘ 2’ shows the ongoing interplay between theory and experiment at work: looking at real data for long enough can force even a theoretical physicist to finally see something!

The second element having an *IP* of order *IO* is the element having nuclear charge *Z* = *IO* + 1. This *IP* is given by

(3) $$IP_{IO,IO+1}=IP_{IO,IO}+\frac{1}{2}\times IP_{1,2}\times IO.$$

Then inserting Eq. (2) with the value *C* = 2,

(4) $$IP_{IO,IO+1}=2\times IP_{1,1}\times IO^{2}+\frac{1}{2}\times IP_{1,2}\times IO.$$

Eq. (4) for *IP_IO_*_,_*_IO_*_+1_makes clear that for *Z* = *IO* + 1 there exists not only a contribution that scales with the quadratic *IO*^2^, but also a contribution that scales with the linear *IO*. The presence of the quadratic term again suggests a physical process involving all of the *IO* electrons being removed. The presence of the linear term suggests a SECOND physical process, involving just the one remaining electron, perhaps a ‘resettling’ into a system of net charge + *IO*

Eq. (4) for *IP_IO_*_,_*_IO_*_+1_ presages the form of the *IP* of order *IO* for the third element that has one. This element has nuclear charge *Z* = *IO* + 2. This *IP* refers, not to Hydrogen, but rather to Lithium, the start of the second period in the PT/PA. It is given by

(5) $$IP_{IO,IO+2}=\frac{1}{2}\times IP_{1,3}\times IO^{2}+\frac{1}{2}\times IP_{1,3}\times IO.$$

This *IP* has the same form as Eq. (4) for *IP_IO_*_,_*_IO_* _+ 1_, but with the coefficient 1 / 2 replacing the coefficient *C* = 2, and *IP*_1,3_ replacing *IP*_1,1_. Now we have two electrons remaining, instead of just one, as in *IP_IO_*_,_*_IO_* _+ 1_. The linear ‘resettling’ term is essentially the same in form, as if only the net positive charge of the ion actually matters, not the individual numbers of protons or electrons.

The fourth element having an *IP* of order *IO* is the one having nuclear charge *Z* = *IO* + 3. The formula for that *IP* refers to Beryllium as well as Lithium:

(6) $$IP_{IO,IO+3}=\frac{1}{2}\times IP_{1,3}\times IO^{2}+(IP_{1,4}-\frac{1}{2}\times IP_{1,3})\times IO.$$

And in fact, Eq. (6) is just a special case of a general formula that applies for *Z* = *IO* + 3 through *Z* = *IO* + 9 or *N*_next_ = *N*_start_ = 3 through *N*_next_ = 9 in:

(7) $$IP_{IO,IO+N_{\text{next}}}=\frac{1}{2}IP_{1,3}IO^{2}+(IP_{1,N_{\text{next}}+1}-\frac{1}{2}IP_{1,3})IO.$$

The 11’th element that has an *IP* of order *IO* is that with nuclear charge *Z* = *IO* + 10. For that atom, and for all atoms in the period *N*_next_ = *N*_start_ = 11 through *N*_next_ = 17, we have

(8) $$IP_{IO,IO+N_{\text{next}}}=\frac{1}{2}IP_{1,11}IO^{2}+(IP_{1,N_{\text{next}}+1}-\frac{1}{2}IP_{1,11})IO.$$

Every subsequent period is like that. We always have:

(9) $$IP_{IO,IO+N_{\text{next}}}=\frac{1}{2}IP_{1,N_{\text{start}}}IO^{2}+(IP_{1,N_{\text{next}}+1}-\frac{1}{2}IP_{1,N_{\text{start}}})IO,$$

where *N*_start_ = 3,11,19,37,55,87,...

The model given here is the simplest and best presently available. Its weakest area is its fit to second-order and third-order *IP*’s for elements in the sixth and seventh periods. Because the model works so well elsewhere, it is natural to scrutinize those points especially closely for clues to the mismatches there. It may be important that the reported second- and third-order *IP*’s there are very close to 2 and 3 times the corresponding first-order *IP*’s. It is possible that the physical process that produced the data was actually different from what was intended: coincident production of multiple atoms each singly-ionized instead of production of a single atom multiply-ionized.

The algebraic model seems to have a simple message to convey. Evidently, the typical *IP* of order *IO* involves THREE physical processes:

1. Removal of *IO* electrons from an atomic system, which is thereby left with net positive charge of +*IO*. The energy required for this process must scale with *IO*^2^. So it has to be the term $\frac{1}{2}IP_{1,N_{\text{start}}}IO^{2}$ in Eq. (9).
2. But before that must come removal of *IO* electrons from the atom’s electron population, which will then be left with *Z − IO* electrons. The energy required for this process must depend on the specific *Z*, which enters Eq. (9) only through the variable *N*_next_. So it has to be the term *IP*_1,*N*_next_+1_*IO* in Eq. (9).
3. There must also be reconstruction/reinstallation of the smaller population of *Z − IO* electrons. As the reverse of destruction/removal, the energy ‘required’ for this process is negative. So it has to be the term $-\frac{1}{2}\times IP_{1,N_{\text{start}}}IO$ in Eq. (9).

The first two *IP*’s of any order *IO* > 1 are not quite typical. The general case is Eq. (9), but for total ionization, *IP_Z,Z_* = *IP_IO,IO_* = 2 × *IP*_1,1_ × *IO*^2^ [Eq. (2)], and for removing *IO*− 1 electrons, thus 1 leaving just one electron, *IP*_*Z*−1,*Z*_ = *IP_IO_*_,_*_IO__+1_* = 2 × *IP*_1,1_ × *IO*^2^+ ½ × *IP*_1,2_× *IO*[Eq. (4)]. Why are the underlined factors 2 instead of 1 / 2? Observe that Hydrogen is exceptional: it has no electron-electron interactions. So only process 1 exists; there is no Process 2 or Process 3. So Hydrogen’s *IP*_1,1_ is deficient in information as a period-start reference for other more normal elements. Observe that the general sum of the absolute coefficients in Eq. (9) is 1 / 2 + 1+|− 1 / 2|=2. This suggests that Hydrogen’s information needs to be scaled up by 2 rather than down by ½ in the *IO*^2^ terms for the first two *IP*s’s of order *IO*.

**Figure 3.**
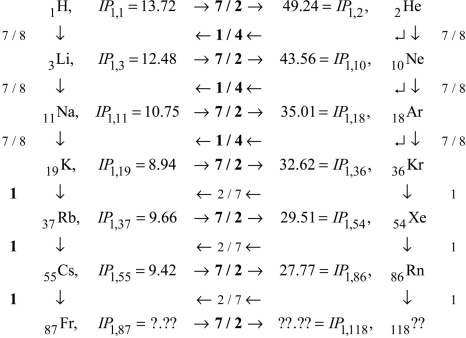
First-order *IP*’s: map of main highways through the periods.

These comments complete the demonstration that first-order *IP*’s contain ALL the information necessary to predict ALL the higher-order *IP*’s via scaling laws. We come now to the problem of the input first-order *IP*’s themselves. As shown on Fig. 2, first-order *IP*’s exhibit consistent rises on periods, and consistent mid-period similarity to Hydrogen. The rise factors are all 7 / 2. After the first three periods, the period maxima are all similar to each other, and the period minima are all similar to each other, and the period mid regions are all similar to Hydrogen. Figure 3 details these facts. Observe that the neighboring *IP*’s are connected to each other by scale factors, such as 7 / 2, 1 / 4, 7 / 8, *etc*. These scale factors correspond to alternate and redundant paths through the data. The path actually used in the excel program that created Fig. 2 is indicated by the scale factors that are displayed in bold font.

The consistency of the rises of 7 / 2 can be appreciated, if not fully understood, as a manifestation of periodicity: all periods in the PT/PA are fundamentally similar. The numerical value 7 / 2 can be understood in relation to the already noted connections between *IP*’s of order 1 and those of higher order *IO* > 1. The expression

(10a) $$IP_{1,2}=\frac{7}{2}IP_{1,1}$$

can be written

(10b) $$IP_{1,2}=2\times IP_{1,1}\times 2\times 1+(0-\frac{1}{2}IP_{1,1})\times 1$$

or

(10c) $$IP_{IO,1+IO}=2\times IP_{1,1}\times (IO+1)\times IO+(0-\frac{1}{2}IP_{1,1})\times IO$$

with *IO* = 1, which for general *IO* resembles Eq. (9) except for the following differences:

1. The factor of 2 instead of the factor 1 / 2, which any reference to Hydrogen requires;
2. The factor (*IO* + 1) × *IO* in place of the *IO*^2^ factor, which is appropriate because Helium has nuclear charge *Z* = 2 = *IO* + 1;
3. The zero in place of to use. *IP*_1,_*_N_*, which is appropriate because there exists no input *IP*_1,2_to use.

All of this suggests that the connections between first *IP*’s of different elements resemble the connections between *IP*’s of different orders. This suggests in turn that all *IP*’s of all orders can ultimately be related back to Hydrogen – the prototypical ‘keystone’ element (Fig. 1).

However, there exist many more details to specify. Within the periods beyond the first one, the rise is nowhere steady; there is a lot of detailed structure in the *IP* plots. On the log scale of Fig. 2, there appear to be straight-line runs interrupted by discontinuities. The straight-line runs are associated with nominal blocks of the traditional angular momentum quantum number *l* [4,5]. Every straight-line run is characterized by:

1. A total rise over the run, and
2. An intercept with the ‘main-highway’ straight line through the period.

The rise appears to be a function of the parameter *N* = 1,2,2,3,3,4,4 that belongs to the period, and the angular momentum quantum number *l* that varies within the period from 0 to *N* – 1. The rise for *l* = 0 is just the full period rise for *N* = 1, and for *N* > 1 it appears to decline with each period. The sequence plotted in Fig. 1 is 1,Ê1 / 2,Ê1 / 3,Ê1 / 4, with 1 / 4 repeated thereafter.

**Figure 4.**
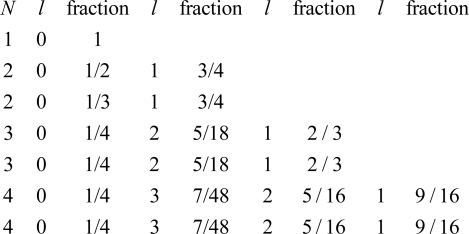
First-order *IP*’s: map of local roads through the periods.

The rise for *l* > 0 offers a lot more data to support a choice of model. The following function was developed for use in Fig. 4 for all *l* > 0:

(11a) incremental rise = total rise ×fraction,

where

(11b) $$\text{fraction}=\left[(2l+1)/N^{2}\right]\left[(N-l)/l\right].$$

The first factor in square brackets is just the ratio of the multiplicity of *l* states, 2(2*l* + 1), to the period length, 2*N*^2^. The second factor, (*N* − *l*) / *l*, captures the real variability in slopes. Figure 4 gives the overall fraction for all *N*,*l* pairs of interest.

The intercepts for *l* = 0 and *l* = 1 are fixed by the period boundaries: the first *l* = 0 point is tied to the period start, and the last *l* = 1 point is tied to the period end. The intercepts for *l* = 2 and *l* = 3 are set at the midpoints of those runs. Because 2(2*l* + 1) is an even number, midpoints fall between elements. The data often shows some type of discontinuity at run midpoints (where the spin quantum number *s* changes sign). For the model, the *IP*’s just above and below these mid points are set equal, making a tiny flat spot on the plotted curves on Fig. 2.

The main conclusion to be drawn from this Section is that a great body of data about *IP*’s, which at first looks to have been created by a random-number generator, is in fact very strictly ordered and predictable.

## 4. Variant Quantum Mechanics

But we do not *really* know what is going on here. The algebraic model as presented is empirical, and not derived from one fundamental theory. It utilizes some variables from traditional QM, namely the angular momentum quantum number *l*, and in a very marginal way the spin quantum number *s*. But QM’s ‘principal’ quantum number *n* is nowhere to be found. Instead there is a very dominant variable *not* from QM; namely, the variable *N* that comes from the periods of the PT/PA. And there is the *M* / *Z* scaling, which is foreign to traditional QM. Clearly, if there is to be a truly theoretical explanation of the *IP* data, there has to be some type of expansion of QM to provide the theory. The basis for an expanded QM lies in an expanded special relativity theory, which is deferred to the next Section. The present Section just summarizes key results of an expanded QM.

**Figure 5.**
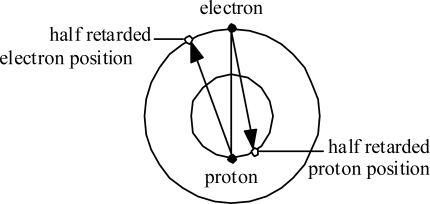
Coulomb force directions within the Hydrogen atom.

Consider first the Hydrogen atom. The electron orbits at radius *r_e_* and the proton orbits at much, much smaller radius *r_p_*. Fig. 5 illustrates in an exaggerated manner how each experiences Coulomb attraction to the ‘half-retarded’ position of the other (as if the Coulomb force vector propagated at speed 2*c* ).

This situation implies that the forces within the Hydrogen atom are not central, and not even balanced. This situation has two major implications:

1. The unbalanced forces mean that the system as a whole experiences a net force. That means the system center of mass (C of M) can move.
2. The non-central individual forces, and the resulting torque, means the system energy can change.

These sorts of bizarre effects never occur in Newtonian mechanics. But electromagnetism is not Newtonian mechanics. In electromagnetic problems, the concepts of momentum and energy ‘conservation’ have to include the momentum and energy of fields, as well as those of matter. Momentum and energy can both be exchanged between matter and fields. ‘Conservation’ applies only to the system overall, not to matter alone (nor to fields alone either).

Looking in more detail, the unbalanced forces in the Hydrogen atom must cause the C of M of the whole atom to traverse its own circular orbit, on top of the orbits of the electron and proton individually. This is an additional source of accelerations, and hence of radiation. It evidently makes even worse the original problem of putative energy loss by radiation that prompted the development of QM. But on the other hand, the torque on the system implies a rate of energy gain to the system. This is a candidate mechanism to compensate the rate of energy loss due to radiation. That is why the concept of ‘balance’ emerges: there can be a balance between radiation loss of energy and torquing gain of energy.

**Figure 6.**
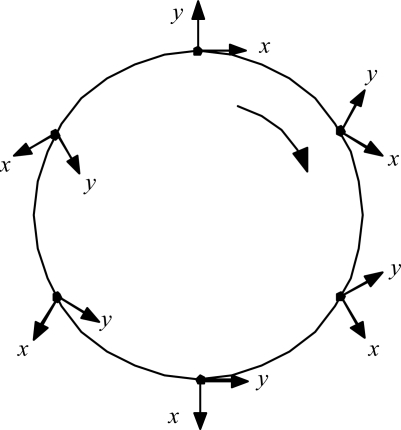
Thomas rotation. When the particle traverses the full circle, its internal frame of reference rotates 180°.

The details are worked out quantitatively as follows. First, ask what the circulation can do to the radiation. A relevant kinematic truth about systems traversing circular paths was uncovered by L.H. Thomas back in 1927, in connection with explaining the then-anomalous magnetic moment of the electron: just half its expected value [8]. He showed that a coordinate frame attached to a particle driven around a circle naturally rotates at half the imposed circular revolution rate. Figure 6 illustrates.

Applied to the old scenario of the electron orbiting stationary proton, the gradually rotating *x*,*y* coordinate frame of the electron meant that the electron would see the proton moving only half as fast as an external observer would see it. That fact explained the electron’s anomalous magnetic moment, and so was received with great interest in its day. But the fact of Thomas rotation has since slipped to the status of mere curiosity, because Dirac theory has replaced it as the favored explanation for the magnetic moment problem. Now, however, there is a new problem in which to consider Thomas rotation: the case of the C of M of a whole Hydrogen atom being driven in a circle by unbalanced forces. In this scenario, the gradually rotating local *x*, *y* coordinate frame of the C of M means that the atom system doing its internal orbiting at frequency Ω_e_ relative to the C of M will be judged by an external observer to be orbiting twice as fast, at frequency Ω′ = 2Ω_e_ relative to inertial space. This perhaps surprising result can be established in at least three ways:

1. By analogy to the original problem of the electron magnetic moment;
2. By construction of Ω′ in the lab frame from Ω_e_in the C of M frame as the power series $\Omega '=\Omega_{\text{e}}\times (1+\frac{1}{2}+\frac{1}{4}+\frac{1}{8}+...)\to \Omega_{\text{e}}\times 2$;
3. By observation that in inertial space Ω′ must satisfy the algebraic relation Ω′ = Ω + ′/2, which implies Ω′ =2Ω_e_.

The relation Ω′ = 2Ω_e_ means the far field radiation power, if it really ever manifested itself in the far field, would be even stronger than classically predicted. The classical Larmor formula for radiation power from a charge *e* (*e* in electrostatic units) is *p_e_* = 2*e*^2^*a*^2^ / 3*c*^3^, where *a* is total acceleration. For the classical electron-proton system, most of the radiation comes from the electron orbiting with $a_{\text{e}}=r_{\text{e}}\Omega_{\text{e}}^{2}$, Ω*_e_* But with Ω′_e_= 2Ω_e_, the effective total acceleration is *a*′ = *a*_e_× 2^2^. With electron-proton total separation nominally *r_e_* +*r_p_*, the Coulomb force is approximately *F*_e_ = *e*^2^/ (*r*_e_ + *r*_p_)^2^, *a*_e_ = *F*_e_ / *m*_e_, and the total radiation power is approximately

(12a) $$P_{\text{R}}=\underline{2^{4}}(2e^{2}/3c^{3})a_{\text{e}}^{\text{2}}=\underline{2^{5}}(e^{6}/m_{\text{e}}^{2})/3c^{3}{\left(r_{\text{e}}+r_{\text{p}}\right)}^{4}.$$

However, that outflow of energy due to radiation is never manifested in the far field because it is compensated by an inflow of energy due to the torque on the system. This is what overcomes the main problem about Hydrogen that was a main driver in the development of QM; namely, that the Hydrogen atom ought to run down due to radiative energy loss.

Generally, the inflow power *P*_T_ = *T*Ω_e_, where *T* is the total torque T =|**r**_e_ × **F**_e_ + **r**_p_ × **F**_p_|, and **r**_e_ × **F**_e_ ≡ **r**_p_ × **F**_p_, so *T*= 2|**r**_e_ × **F**_e_ |. With two-step light, the angle between **r**_e_ and **F**_e_ is *r*_p_Ω_e_/2*c* = (*m*_e_/ *m*_p_)(*r*_e_Ω_e_ / 2*c*). So the torque *T* = (*m*_e_/*m*_p_)(*r*_e_Ω_e_ / *c*)[*e*^2^/ (*r*_e_ + *r*_p_)] and the power

(12b) $$P_{\text{T}}=(m_{\text{e}}/m_{\text{p}})(r_{\text{e}}\Omega_{\text{e}}^{\text{2}}/c)\left[e^{2}/(r_{\text{e}}+r_{\text{p}})\right]=(e^{4}/m_{\text{p}})/c{\left(r_{\text{e}}+r_{\text{p}}\right)}^{3}.$$

Now posit a balance between the energy gain rate due to the torque and the energy loss rate due to the radiation. The balance requires *P*_T_ = *P*_R_, or

(12c) $$(e^{4}/m_{\text{p}})/c{\left(r_{\text{e}}+r_{\text{p}}\right)}^{3}=(2^{5}e^{6}/m_{\text{e}}^{2})/3c^{3}{\left(r_{\text{e}}+r_{\text{p}}\right)}^{4}.$$

This equation can be solved for *r_e_* +*r_p_* :

(13a) $$r_{\text{e}}+r_{\text{p}}=32m_{\text{p}}e^{2}/3m_{\text{e}}^{2}c^{2}=5.5\times 10^{-9}\text{ cm}.$$

Compare this value to the accepted value *r_e_* + *r_p_* = 5.28 × 10^−9^cm The match is fairly close, running just about 4% high. That means the concept of torque versus radiation does a fairly decent job of modeling the ground state of Hydrogen.

The result concerning the Hydrogen atom invites a comment on Planck’s constant *h*, which is generally presumed to be a fundamental constant of Nature. In conventional QM, *r*_e_ +*r*_p_ is expressed in terms of *h*:

(13b) $$r_{\text{e}}+r_{\text{p}}=h^{2}/4\pi^{2}\mu e^{2}.$$

Here μ is the so-called ‘reduced mass’, defined by μ^−1^ = *m*_e_^−1^ + *m*_p_^−1^. Using μ ≈ *m*_e_ in (13b) and equating (13b) to (13a) gives

(14) $$h\approx \frac{\pi e^{2}}{c}\sqrt{128m_{\text{p}}/3m_{\text{e}}}.$$

This expression comes to a value of 6.77 × 10^−34^ Joule-sec, about 2% high compared to the accepted value of 6.626176 × 10^−34^ Joule-sec. Is this result meaningful? To test it, a more detailed analysis accounts more accurately for ‘sin’ and ‘cos’ functions of the small angle *r*_p_Ω_e_/2*c*, here represented by the small angle itself, and by unity. That exercise makes the estimate of *h* more accurate too, and suggests that the model is indeed meaningful, and that Planck’s constant need not be regarded as an independent constant of Nature.

The analysis so far is for the ground state of Hydrogen. To contribute to a covering theory for QM, that analysis has to be extended, first to cover trans-Hydrogenic atoms, and then to cover the so-called ‘excited states’ of Hydrogen, and the trans-Hydrogenic atoms, and even molecules.

The first concept for creating extensions is to replace the proton in Hydrogen with other nuclei. This replacement immediately gives the reason for the *M* / *Z* scaling used throughout this paper. With replacement, the subscript p for proton changes to *Z*. Eqs. (12a) and (12b) are both scaled by *Z*^2^, and (12b) is additionally scaled by 1/ *M*. As a result, (13a) changes to *r*_e_ + *r_Z_* = M(*r_e_*+*r_p_*). The electron energy in the Hydrogen case is *E*_H_ = e^2^/ (*r*_e_ +*r*_p_); for the element *Z* case, the *e*^2^ changes to *Ze*^2^, so overall, the single-electron energy changes to

(15) $$E_{Z}=\hat{E}Ze^{2}/M(r_{\text{e}}+r_{Z})=(Z/M)E_{\text{H}}.$$

If it weren’t for neutrons, the scale factor *Z* / *M* would be unity. But because of neutrons, *Z* / *M* varies from 1 for Hydrogen, immediately to 0.5 for Helium, and eventually to 0.4 for the heaviest elements we presently know about. So in order to put the *IP* data for different elements onto a common basis, we must remove the *Z* / *M* factor from raw data by scaling with its inverse *M* / *Z*.

The second concept for creating extensions is to replace the single electron and single proton in Hydrogen with multiple electrons and multiple protons (with neutrons too), charges of each sign bound in coherent subsystems called ‘charge clusters’. In the journal Galilean Electrodynamics, we have occasionally had reports and commentary about the apparently incomprehensible phenomenon of electrons clustering together [9–11]. The phenomenon is widely known; related literature cited in the third of those references is quite extensive, and some of it appears in the most widely circulated physics journals.

The idea of charge clusters suggests a new interpretation of ‘excited’ states for Hydrogen. The conventional idea involves an electron teetering in an upper ‘shell’, ready to fall back to a lower ‘shell’. But the present simple two-body analysis of Hydrogen does not allow anything so complicated. The simple torque *vs*. radiation balance has only one low-speed solution, corresponding to the ground state. That means the term ‘excited state’ cannot describe a condition of a single Hydrogen atom. So it has to describe a system of multiple Hydrogen atoms.

Support for an excitation model based on multiple atoms comes from the known fact that light emission is always a little bit laser-like, in that photons are emitted, not as singletons, but rather in bursts [12]. This behavior suggests that atoms become excited not as singletons, but as groups. So suppose that ‘excitation’ of Hydrogen up to state *n* actually involves *n* = *n*_H_ Hydrogen atoms all working together in a coherent way. In particular, suppose that the *n*_H_ electrons make a negative cluster, and the *n*_H_ protons make a positive cluster, and the two clusters together make a scaled-up Hydrogen super-atom.

The replacement of single charges with charge clusters must affect both the radiation energy loss rate and the torquing energy gain rate, and the balance between them. Every factor of *e* and every factor of *m*_e_ or *m*_p_ scales by *n*_H_. Starting from (12a) for the radiation, one finds that the energy loss rate scales by *n*_H_^4^. Starting from (12b) for the torquing, one finds that the energy gain rate scales by *n*_H_^3^. The solution radius for system balance therefore scales as *r*_e_ + *r*_p_ → *r*_*n*_H__ = *n*_H_ (*r*_e_ + *r*_p_). [Note: if this multi-atom model captures the real behavior behind atomic excitation, and if one attempts to model that behavior in terms of a single atom with discrete radial states identified with a principal quantum number *n*, then the radial scaling has to be *r*_1_ → *r_n_* = *n*^2^*r*_1_, as is seen in standard QM.].

The overall system orbital energy then scales as *E*_1_ → *E*_*n*_H__ = *n*_H_^2^*E*_1_ / *n*_H_ = *n*_H_*E*_1_. This energy result is exactly the same as the orbital energy of *n*_H_ *separate* atoms *not* clustered together in a super atom. The implication is that when the system disintegrates, the energy that exits as photons does *not*, as is generally believed, correspond to an orbit around the nucleus. It is instead the positive energy required to form the charge clusters. If any kind of ‘orbit’ is involved, it is an orbit, not around the nucleus, but rather internal to the charge cluster. This is a completely novel view of excitation.

Spectroscopic data indicates that the energy required to bring the *n*_H_^th^ Hydrogen atom from complete separation to complete integration into an existing super atom of *n*_H_ − 1 atoms, thus forming a super atom of *n*_H_ atoms, is |*E*_1_| [(*n*_H_−1)^−2^−*n*_H_^−2^]. The inverse squares can be understood as follows. The radial scaling *r*_*n*_H__ = *n*_H_ (*r*_e_ + *r*_p_) suggests that all linear dimensions scale linearly with *n*_H_. If so, the volume of the clusters scales as *n*_H_^3^. The number density of charges in clusters therefore scales as *n*_H_/*n*_H_^3^ = *n*_H_^−2^ The positive energy locked in the pair of clusters therefore depends on the number density in the clusters. This is something like having energy proportional to pressure, as is seen in classical thermodynamics.

The charge-cluster model for excitation suggests that there ought to be some similarity between Hydrogen in its first excited state (*n*_H_ = 2) and a Hydrogen dimer molecule. Both have two electrons; both are favored, just like a Helium atom is favored. The preference for a two-atom excited state would explain why the spectrum of Hydrogen so strongly features transitions that terminate, not with the ground state, but rather with the first excited state.

The idea of charge clusters suggests that if ‘shells’ of any kind exist in trans-Hydrogenic atoms, then they are probably not centered on the nucleus, but instead nested in an electron charge cluster. The innermost shell must have two electrons. The number of electrons in remaining shells has to increase with the radius of shells, keyed to *N*. Electron pairs have to occupy increasingly greater numbers of zenith positions in relation to the plane of the central two-body system, in a way that matches the behavior of the traditional quantum number *l*. But it is not really the traditional *l* here, since the charge cluster is a completely different vision of atomic structure.

One general conclusion to be drawn from this Section is that charge clusters are important, and possibly ubiquitous in atoms. But explaining how charge clusters can even exist requires the same sort of information as does explaining the ‘half-retarded’ directionality notion at the beginning of this Section; namely, an expanded SRT. This comes next.

## 5. Expanded Special Relativity Theory

Einstein [13,14] elevated an idea that had emerged from study of Maxwell to the status of a founding Postulate for Special Relativity Theory (SRT). Maxwell had the free-space electric permittivity ε_0_ and magnetic permeability μ_0_, which together imply a light speed *c*. Einstein’s famous ‘Second Postulate’, asserted this light speed to be the same constant for all inertial observers, independent of any particular circumstance, such as source motion.

Inasmuch as SRT is founded on Maxwell’s theory, and Maxwell’s theory cannot handle the Hydrogen atom, SRT is unlikely ever to be fully compatible with QM. Einstein was involved in the development of QM, through his Nobel-Prize winning work on the photoelectric effect, but he was not fond of QM, and in later years did not work so much on it. Instead, he mainly went back to SRT, embraced the Minkowski tensor formulation for it, and exploited the metric tensor therein to develop General Relativity Theory (GRT).

GRT has the same fundamental character as Maxwell’s theory: it is a field theory, and as such, it is not designed for something so complicated as a two-body problem. It is the extreme opposite to Newton’s point-particle theory, which excels on the two-body problem. Late in life, Einstein wrote to his friend M.A. Besso about his misgivings concerning field theories:

I consider it quite possible that physics cannot be based on the field concept, *i.e.*, on continuous structures. In that case *nothing* remains of my entire castle in the air, gravitation theory included, [and the] rest of physics.

Acknowledging such doubts is, I believe, the mark of a truly great scientist. Einstein’s present-day followers usually do not harbor such doubts.

But SRT has produced an extensive literature about ‘paradoxes’, especially featuring twins, clocks, trains, meter sticks, or barns, or spinning disks, *etc*. So there have always been researchers questioning Einstein’s Second Postulate, and evaluating alternatives to it. Ritz [15] was an early, but unsuccessful, example. Later, in the 1950’s, began the work of P. Moon, D. Spencer, E. Moon, and many of Spencer’s students [see [16–18] and additional references cited therein]. Their work has been successful in producing a lot of very interesting results, if not in garnering all the recognition it really deserves.

The key Moon-Spencer-Moon *et al*. idea was a propagation process with continuing control by the source, even after the initiating ‘emission’ event, so that the light moves away from the source at speed *c* relative to that source, however arbitrarily the source itself may be moving. (This is *not* the Ritz postulate, which had the light moving at velocity **c** +**V**, where **V** was the velocity vector of the source at the moment of emission, and **c** is the velocity vector of the light if it had come from a stationary source at that moment.)

In any event, continuing control by the source implies that ‘light’, whatever it is, has a longitudinal extent (Of course! Light possesses wavelength, does it not?), and the longitudinal extent is expanding in time. That expansion naturally raises the question: exactly what *feature* of the expanding light packet is it that moves at speed *c* relative to the source? The tacit hypothesis of Moon-Spencer-Moon *et al*. is that the *c*-speed part is the leading tip of the light packet. It then follows that when a receiver is encountered, the entire longitudinal extent of the light packet must collapse instantly to the receiver. That means the trailing tail of the light packet must snap into the receiver at infinite speed. The infinite speed might be unacceptable for Einstein true believers, but maybe not for QM true believers.

My own work [19,20] follows the Moon-Spencer-Moon *et al*. lead, with one conceptual addition; namely, that the speed *c* relative to the source characterizes, not the leading tip of the light packet, but rather the mid point of the light packet. That means the leading tip must move relative to the source, not at *c*, but rather at 2*c*. [A 2*c* anywhere is probably shocking to Einstein true believers, but maybe not so shocking as an infinite speed would be.]

This variation on the Moon-Spencer-Moon *et al*. theme allows symmetry between light emission and absorption. The leading tip of the light packet reaches the receiver in half the time for propagation at *c*, so there is time left for a completely symmetric absorption process, wherein the mid point of the light packet travels at speed *c* relative to the receiver, however arbitrarily that may move. That idea then means the tail end reels in at speed 2*c* relative to the receiver.

**Figure 7.**
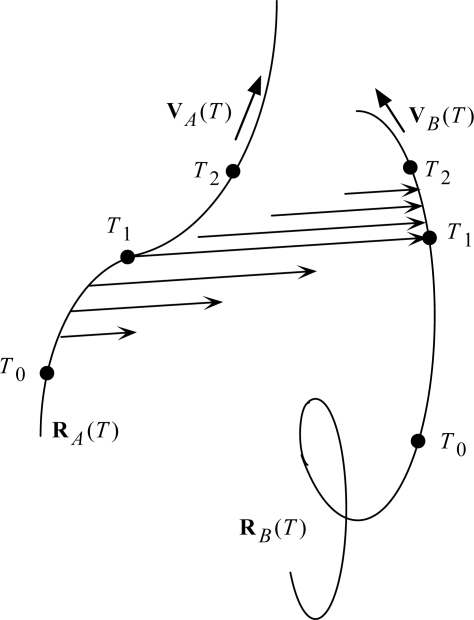
Illustration of Two-Step Light propagation.

The revised light postulate is what I have called ‘Two-Step Light’. It is illustrated in Fig. 7. The *T*’s are Universal Times: *T*_0_ at the beginning of the scenario, *T*_1_ at the mid point, and *T*_2_ at the end. Particle *A* is the source, and particle *B* is the receiver (one of possibly many candidate receivers, selected by the accidental collision with the expanding light arrow at *T*_1_).

The mid points of the light arrows may be said to resemble the Moon-Spencer-Moon *et al*. favored postulate in the expansion phase of the scenario, and then with the Einstein postulate in the collapse phase of the scenario. How can light do all that? Stay in contact with a moving source? Switch control to a moving receiver? Stay in contact with a moving receiver? At this point, I must follow Newton, who answered all such ‘how’ questions with the phrase *hypothesis non fingo*. My first job is just to work out the implications of the Two-Step Light Postulate. It is a straightforward task, involving just algebra. It has been detailed in [19,20]; here I shall just summarize results.

Consider the problem of processing data consisting of successive light signals from a moving source in order to estimate the speed *V* of that source. If the light propagates according to the Two-Step process, but the data gets processed under the assumption of the one-step Einstein postulate, then there will be a systematic error to the estimate. In fact, the estimate turns out to be:

(16) $$v=V/(1+V^{2}/4c^{2}).$$

The estimate *v* is always less than *V*, and in fact is limited to *c*, which value occurs at *V* = 2*c*. Thus *v* has the property that is characteristic of any observable speed in Einstein’s SRT. The obvious implication is that *v* is an Einsteinian speed, whereas *V* is a Galilean speed.

One is obviously invited to look also at a related construct

(17) $$V^{↑}=V/(1-V^{2}/4c^{2}).$$

The superscript ↑ is used to call attention to the fact that *V*^↑^ has a singularity, which is located at *V* = 2*c*, or *v* = *c*. That is, *V*^↑^ has the property of the so-called ‘proper’ or ‘covariant’ speed. Interestingly, past the singularity, *V*^↑^ changes sign. This behavior mimics the behavior that SRT practitioners attribute to ‘tachyons’, or ‘super-luminal particles’: they are said to ‘travel backwards in time’. The sign change is a mathematical description, while the ‘travel backwards in time’ is a mystical description.

The relationships expressed by (16) and (17) can be inverted, to express *V* in terms of *v* or *V*^↑^. The definition *v* = *V* / (1 + *V*^2^ / 4*c*^2^) rearranges to a quadratic equation (*v* / 4*c*^2^)*V*^2^ − *V* + *v* = 0, which has solutions

(18a) $$V=\frac{1}{v/2c^{2}}\left(+1\pm \sqrt{1-v^{2}/c^{2}}\right).$$

Multiplying numerator and denominator by $\left(+1\text{m}\sqrt{1+V^{↑2}/c^{2}}\right)$ converts these to the form

(18b) $$V=v/\frac{1}{2}\left(1\text{m}\sqrt{1-v^{2}/c^{2}}\right),$$

which makes clear that for small *v*, *V* has one value much, much larger than *v*, and another value essentially equal to *v*.

Similarly, the definition *V*^↑^ = *V* / (1 − *V*^2^ / 4*c*^2^) rearranges to a quadratic equation (− *V*^↑^ / 4*c*^2^)*V*^2^ − *V* + *V*^↑^ = 0, which has solutions

(19a) $$V=\frac{1}{-V^{↑}/2c^{2}}\left(+1\pm \sqrt{1-V^{↑2}/c^{2}}\right).$$

Multiplying numerator and denominator by $\left(+1\text{m}\sqrt{1+V^{↑2}/c^{2}}\right)$ converts these to the form

(19b) $$V=V^{↑}/\frac{1}{2}\left(1\text{m}\sqrt{1-V^{↑2}/c^{2}}\right).$$

which makes clear that for small *V*^↑^, *V* has one value much larger in magnitude than *V*^↑^ (which is negative there), and another value essentially equal to *V*^↑^.

To see that *v* and *V*^↑^ are not only qualitatively *like* Einsteinian speed and covariant speed, but in fact quantitatively *equal* to them, one can do a bit more algebra. Substitute (18b) into (17) and simplify to find

(20a) $$V^{↑}=\text{m}v/\sqrt{1-v^{2}/c^{2}},$$

which is the definition of covariant speed familiar from SRT, made slightly more precise by inclusion of the minus sign for situations beyond the singularity.

Similarly, substitute (19b) into (16) and simplify to find

(20b) $$v=mV^{↑}/\sqrt{1+V^{↑2}/c^{2}},$$

which is again a relationship familiar from SRT, made slightly more precise by inclusion of the minus sign for situations beyond the singularity.

**Figure 8.**
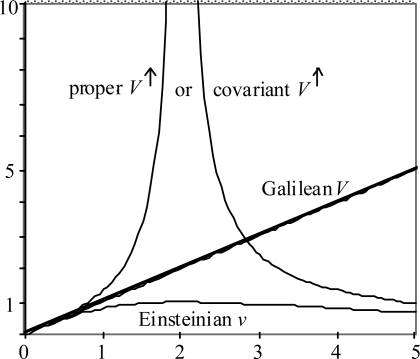
Numerical relationships among three speed concepts.

The information contained in Eqs. (16)–(20a,b) is displayed graphically in Fig. 8. Both plot axes denote multiples of nominal light speed *c*. Galilean particle speed *V* is the independent variable. To save space beyond the singularity, where *V*^↑^ goes negative, it is the absolute value of *V*^↑^ that is plotted.

Speed can be seen as a proxy for many other interesting things in SRT, like momentum, relativistic mass, *etc*. Observe that with only two speed concepts, SRT only can offer only two speed relationships, whereas with three speed concepts, Two Step Light offers six speed relationships. This constitutes three times the information content. This is what makes Two Step Light a ‘covering theory’ for SRT. Two Step Light offers additional opportunities for explaining all the interesting things in SRT.

The word ‘interesting’ is sometimes a euphemism for the word ‘paradoxical’. The fact that Galilean speed *V* is missing from the language of SRT means that Einsteinian speed *v* gets conflated with Galilean speed *V* in SRT. Any conflation of different physical concepts causes confusion and misinterpretation of both theoretical and experimental results. That is why the literature of SRT contains so much discussion of ‘paradoxes’. But there are no paradoxes in physical reality, and there are none in Two Step Light theory. To illustrate this point, consider one rather obscure but very important case. The established Liènard-Wiechert potentials and fields [21,22] for rapidly moving sources have a most paradoxical property.

Expressed in Gaussian units [23], the Liènard-Wiechert scalar and vector potentials are:

(21a) $$\Phi (x,t)=e{\left[1/\kappa R\right]}_{\text{retarded}}\text{ and }A(x,t)=e{\left[\beta /\kappa R\right]}_{\text{retarded}},$$

where κ = 1 − ngβ, with β being source velocity normalized by *c*, and **n** = **R** / *R* (a unit vector), and **R** = **r**_observer_(*t*) − **r**_source_(*t* − *R* / *c*) (an implicit definition for the terminology ‘retarded’). The Liènard-Wiechert fields expressed in Gaussian units are then

(21b) $$E(x,t)=e{\left[\frac{\left(n-\beta )(1-\beta^{2}\right)}{\kappa^{\text{3}}R^{2}}+\frac{n}{c\kappa^{\text{3}}R}\times \left(\left(n-\beta \right)\times \frac{d\beta }{dt}\right)\right]}_{\text{retarded}}\text{and }B(x,t)=n_{\text{retarded}}\times E(x,t).$$

The 1/*R* fields are radiation fields, and they make a Poynting vector that lies along **n**_retarded_:

(21c) $$P=E_{\text{radiative}}\times B_{\text{radiative}}\hat{E}=E_{\text{radiative}}\times (n_{\text{retarded}}\times E_{\text{radiative}})\hat{E}=E_{\text{radiative}}^{2}n_{\text{retarded}}.$$

But the 1/*R*^2^ fields are Coulomb-Ampère fields, and the Coulomb field does not lie along **n**_retarded_ as one might naively expect; instead, it lies along (**n** − **β**)_retarded_.

Consider the following scenario, designed specifically for an instructive exercise in *reductio ad absurdum*. A source executes a motion comprising two components: **1)** inertial motion at constant **β**, plus **2)** oscillatory motion at small amplitude and high frequency, so that there exists a small velocity Δ**β** _retarded_ and a not-so-small acceleration *d*Δβ/*dt*|_retarded_. Observe that the radiation and the Coulomb attraction/repulsion come from different directions. The radiation comes along n_c_from the retarded source position, but the Coulomb attraction/repulsion lies along (**n** − **β**) _retarded_, which is basically (**n**_retarded_)_projected_, and lies nearly along **n**_present_. This behavior seems peculiar. retarded, Particularly from the perspective of modern Quantum Electrodynamics (QED), all electromagnetic effects are mediated by photons – real ones for radiation and virtual ones for Coulomb-Ampere forces. How can these so-similar photons come from different directions?

Two-Step Light theory resolves the directionality paradox inherent in the Liènard-Wiechert fields. Because of the various 2*c*’s in the mathematics, the radiation changes to direction **n**_retarded_ changes to **n**_half retarded_, and the Coulomb attraction/repulsion direction (**n**_retarded_)_projected_ changes to (**n**_retarded_)_half projected_. These two directions are now physically the same; namely the source-to-receiver direction at the mid point of the scenario, *i.e.***n**_mid point_. The potentials and fields become:

(22a) $$\Phi (x,t)=e{\left[1/R\right]}_{\text{mid point}}\text{ and }A(x,t)=e{\left[V/cR\right]}_{\text{mid point}}$$

and

(22b) $$\text{E}(x,t)=e{\left[\frac{n}{R^{2}}+\frac{n}{cR}\times \left(n\times \frac{dV}{cdt}\right)\right]}_{\text{midpoint}}\text{ and }B(x,t)=n_{\text{mid point}}\times E(x,t)$$

so

(22c) $$P=E_{\text{radiative}}\times B_{\text{radiative}}\hat{E}=E_{\text{radiative}}\times (n_{\text{mid point}}\times E_{\text{radiative}})\hat{E}=E_{\text{radiative}}^{2}n_{\text{mid point}}.$$

Observe that the Coulomb attraction or repulsion is now aligned with the direction of the radiation propagation.

**Figure 9.**
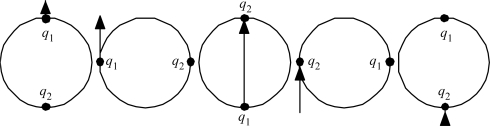
Attraction between two like charges orbiting at superluminal speed *V* = π*c*.

Applied in the Hydrogen atom, Eq. (22b) creates the non-central forces illustrated in Fig. 5. Furthermore, it allows a mechanism that can explain charge clusters. As is emphasized by Fig. 8, there is no limitation on Galilean speed *V*. It can exceed *c*. Figure 9 illustrates a case where two like charges are orbiting each other at speed *V* = π*c*. Because of the ‘mid-point’ feature of (22b), the half-retarded Coulomb ‘repulsion’ between the two charges actually works as attraction.

Fig. 9 just constitutes a ‘proof of existence’: there do exist circumstances in which like charges attract. More detailed analysis, beyond the scope of the present paper, shows that the lowest-speed circumstance that actually satisfies all requirements for stability is not *V* = π*c*, but rather *V* = 3π*c*. And then there is a whole series of higher-speed solutions at higher multiples of πc :7πc, Ê1 1π*c*,Ê1 5π*c*...*etc*.

The main conclusion to be drawn from the present Section of the present paper is that the extended SRT gives two key ingredients for the variant QM: **1)** non-central forces, and hence the possibility for balance between radiation and torquing in the Hydrogen atom, and **2)** the possibility for reversal of Coulomb repulsion into Coulomb attraction, and hence the possibility for explaining the observed existence of charge clusters, here imputed to exist inside trans-Hydrogenic atoms.

## 6. Conclusions

The main conclusions to be drawn from this paper overall are:

1. An important factor presently limiting the development of both physics and chemistry is Einstein’s Second Postulate concerning light speed. We do not have to retain that Postulate. We can consider other postulates instead, and adopt another one if it works better. For example, we can adopt Two Step Light. In that case, what comes out is a covering theory for Einstein’s SRT. Since it contains SRT, researchers who are happy with SRT need not sacrifice anything. But researchers who need something more can perhaps find something they need in Two Step Light.
2. For example, expanding SRT allows one to adopt an approach for understanding atoms that is completely different from traditional QM. We need not postulate the value of Planck’s constant, or the nature of its involvement in the mathematics of ‘probability’ waves, *etc*. Planck’s constant can be an output *from*, rather than an input *to*, the variant theory for atoms. The variant approach resembles the early Thomas-Fermi approach, and the more recent density-functional approaches, in the sense that it seeks a universal solution, particularized to individual elements by scaling laws. But it differs from those other approaches, in that it does not use a spatial density function, or involve any spatial integration to estimate any values of any variables. All it uses is algebra.
3. The algebraic QM supports an algebraic model for ionization potentials, *IP*’s. The model is synoptic: it extends to all possible elements and all possible ionization orders. Its information could be very important and useful. For example, there exist some really ‘bad actors’ among the known elements - think of Plutonium, Polonium, Thallium, or Uranium. There are also some very short-lived elements beyond Uranium. Supposing that there exists some good reason to know more about these dangerous and difficult elements, it is a good thing to have a single, safe and reliable model, instead of many dangerous and difficult experiments, to estimate all their ionization potentials.
4. Beyond that, it is to be hoped that detailed knowledge about *IP*’s will translate into quantitative knowledge about chemical bonds. Here it is even more important to have the algebraic model. Look again at Table 1: it rapidly comes to molecules with many hundreds of electrons, and molecules of interest in the real world are often very much larger still. There is a real prospect that analyzing them will require *IP*’s well beyond those that experimental data provides. The algebraic model is a viable way to estimate missing information like that.
5. Some surprises emerge from the *IP* exercise. For example, the algebraic model seems to conflict with the prescriptions of the Rydberg constant *R*, familiar from spectroscopy, and related to *IP*’s by a conversion of units from eV’s to inverse wavelength. The *R* is said to scale for different elements according to the factor *Z*^2^/ (1 +*m_e_*/*m*_nucleus_). The algebraic model has no *Z*^2^, and its nuclear mass dependence is much stronger − 1/*M*, where *M* is the nuclear mass number – implying interesting isotope effects yet to be investigated. It seems likely that the standard *Z*^2^/ (1 + *m*_e_/*m*_nucleus_) scale factor was just an early guess, offered before spectroscopy was so well developed, or so many *IP*’s had been measured. It now lies fossilized in the pedagogical literature, and needs to be dug out.
6. For another example, the algebraic model gives a prominent role to terms in squared ionization order, *IO*^2^. This raises a subtle point. When one speaks of ionization at order *IO*, should one imagine removal of one more electron from a previously prepared ion of charge +*IO* − 1? Or should one imagine removal of *IO* electrons all at once? Some may favor the former vision because it fits with theory we have been taught. But having seen all the data, with its prominent *IO*^2^ scaling, I now believe the latter vision.
7. Some loose ends remain for future resolution. For example, there is not yet a satisfactory explanation for the parameter *N* that occurs in *IP* estimates. It does not come from traditional QM, and I have not shown so far that it comes from extended QM. For now, *N* remains a mysterious gift from the PT/PA.
8. Charge clusters will probably play a role in explaining *N*. Recall the formula (1) for the lengths of periods; *L* = 2*N*^2^ for *N* = 1,2,2,3,3,4,4. The ‘2’ is understandable as a reference to electron pairs, like ‘Cooper pairs’ familiar from solid-state physics. The *N*^2^ can be understood as a reference to a cluster of electrons that is spherical in shape, with constant electron density over radius. The mystery that remains is the repetition indicated by *N* = 1,2,2,3,3,4,4.
9. I have not detailed how *IP*’s figure into chemical bond strengths. Presumably the story will involve the *IP*’s of the elements in a molecule, the *IP*’s of their noble-gas reassignments, and some *Z*-dependent scaling. But revealing the specifics seems to require a new data-mining exercise more extensive than the one reported here for the *IP*’s alone.
10. The concept concerning chemical bonds that this paper offers is holistic: Propositions 1 and 2 speak about molecules overall, and not about individual atom-to-atom bonds. Those Propositions are here offered for future testing and possible refinement or replacement.

## 7. Notes Added in Proof

In response to Propositions 1 and 2, a reviewer pointed out that oxygen, ozone, and nitrous oxide do possess ‘all-neon’ configurations. This is indeed true, when ‘bonds’ are imagined as electrons ‘shared’ between two atoms, and those shared electrons are ‘double counted’, so that O + O = 8 + 8 = 16 becomes 20 = 10 + 10, or O + O +O = 8 +8 + 8 = 24 becomes 30 = 10 + 10 + 10, or N + N + O = 7 + 7+ 8 = 22 becomes 30 = 10 + 10 + 10. Propositions 1 and 2 do not allow double counting, and for that reason, they identify oxygen, ozone, and nitrous oxide as being quite different from the many other molecules that they identify as ‘stable’. My thanks go to the reviewer for raising this issue.

Guest Editor Prof. Dr. Mihai V. Putz called my attention to his related works [24–26]. Ref. [24] reveals, like the present work does, energy increments that are linear and quadratic in electron numbers. Reaction energies of ‘hard acid’ (H^+^) and ‘soft acid’ (HO^+^) with ‘hard base’ (OH^−^) and ‘soft bases’ (many kinds exist) are studied in [25]. They, and other reaction energies, must be related to the *IP* data here codified in terms of the PT/PA. Trends across the PT/PA are addressed for electronegativity and hardness in [26]. I believe there exists much data here to be further mined!

Dr. Putz also recalled for the author the efforts of Lois de Broglie and his intellectual descendants David Bohm and Jean-Pierre Vigier to rewrite quantum mechanics in a less mysterious way, by introducing the concept of ‘pilot waves’, within which a point particle would travel, a bit like a surfer. There does exist symmetry between that work and the effort described here to rewrite special relativity theory in a less mysterious way; in this case, by introducing an elastic boundary condition for light, a sort of expanding/contracting ‘water balloon’, within which light waves would be confined. By releasing energy, the source would set in motion the first phase front - call it the ‘primary’ one. That primary wave front would then induce other wave fronts, ahead of and behind itself, filling the ‘balloon’ end to end. Individual phase fronts would travel at speed *c*; ends of the boundary ‘balloon’ would travel at 0 and 2*c* in expansion, or 2*c* and 0 in contraction. In both the ‘pilot-wave’ model for particles and the ‘water balloon’ model for light, something of importance lies at the center of the imagined structure: the point particle at the center of the pilot waves, or the primary wave front at the center of the water balloon. I am pleased to have this symmetry now articulated thanks to interaction with Dr. Putz.

## Figures and Tables

**Table 1.** Electron redistributions in relatively stable molecules to give to all of the atoms an electron count equal to that of a noble gas, or else zero.

Name	Chemical formula	Electron contributions	Electron redistributions
Ammonia	NH_3_	N: 7, H’s: 1 each total 10	N : 10, H’s: all 0 total 10
Sodium hydroxide	NaOH	Na : 11, O: 8, H : 1 total 20	Na : 10, O: 10, H : 0 total 20
Potassium carbonate	K_2_CO_3_	K’s: 19 each, total 38	K’s: 18 each; total 36
		O’s: 8 each, total 24	O’s: 10 each, total 30
		C: 6; total 68	C: 2; total 68
Bornyl acetate	CH_3_CO_2_C_10_H_17_	C’s: 6 each, total 72	C’s: 6@2, 6@10; total 72
		H’s: 1 each, total 20	H’s: 8@2, 8@0, total 16
		O’s: 8 each, total 16	O’s: 10 each, total 20
		total 108	total 108
Lead acetate	(CH_3_CO_2_)_2_Pb · 3H_2_O	C’s: 6 each, total 24	C’s: 2 each: total 8
		H’s: 1 each, total 12	H’s: 5@2, 7@0, total 10
		O’s: 8 each, total 56	O’s: 10 each, total 70
		Pb: 82; total 174	Pb: 86; total 174
Calcium stearate	(C_17_H_35_CO_2_)_2_Ca	C’s: 6 each; total 216	C’s: 18@10, 18@2, total 216
		H’s: 1 each, total 70	H’s: 38@0, 32@2, total 64
		O’s: 8 each, total 32	O’s: 10 each, total 40
		Ca : 20 total 338	Ca : 18; total 338
